# Cooperation and conflict in romantic partners’ personal projects: the role of life domains

**DOI:** 10.1007/s12144-022-02813-9

**Published:** 2022-03-01

**Authors:** Orsolya Rosta-Filep, Viola Sallay, Noémie Carbonneau, Tamás Martos

**Affiliations:** 1 Doctoral School, Semmelweis University, Budapest, Hungary; 2 Institute of Psychology, University of Szeged, Szeged, Hungary; 3 Department of Psychology, Université du Québec à Trois-Rivières, Trois-Rivières, Quebec Canada

**Keywords:** Personal projects, Goal content, Romantic partners, Relational conflict, Cooperation; dyadic analysis

## Abstract

Personal projects represent a person’s pursuits in different life domains. The present study examines the orientations of adults’ personal projects and how these orientations are embedded in the dynamics of romantic relationships. Cross-sectional data from 249 married or cohabitating Hungarian heterosexual couples were collected (mean age 42 ± 10.76 and 39.64 ± 10.21 years for male and female partners, respectively). An adapted version of the Personal Project Assessment procedure was completed by both partners individually. Four of their chosen projects were evaluated based on perceived cooperation and conflict regarding these projects and other predefined aspects. First, after applying a person-oriented approach, four meaningful content domains emerged from the thematically coded data using cluster analysis: (1) Practical, (2) Work-Life Balance, (3) Relationships, and (4) Learning and Growth orientations. For both genders, people with Learning and Growth orientation were younger than those with Practical orientation, and among women, the Work-Life Balance orientation group was older. Second, we linked the content domains to relationship experiences on the dyadic level. Both partners with Learning and Growth orientation goals perceived less cooperation. Female partners whose spouses had Work-Life Balance or Learning and Growth orientation goals perceived less conflict regarding their own goals. Overall, Learning and Growth-oriented goals can be considered more distant from the dynamics of romantic relationships because they involve fewer joint experiences and less cooperation and conflict.

## Introduction

Peoples’ daily lives are centered around multiple goals they are trying to accomplish: they work to get promoted, organize their family holiday, and eat more healthy. By continuously investing time and energy into their personal goals, they actively shape the trajectory of their life in the long run. Goals are contextually sensitive dynamic units of self-regulation, linking stable personality traits and specific behavioral actions in response to the environmental possibilities and challenges (Little, 2001; McAdams, 1995).

Even though goals are individual units, they are also embedded in the socioecological context containing cultural norms and personal relationships (Little, 2015). Throughout the adult life span, stable romantic relationships combine both normative and personal contexts: while commitment to a romantic partner is a normative developmental task itself (Arnett, 2000; Erikson, 1950; Havighurst, 1980), partners also share their everyday life for a considerable period, they face similar contextual challenges and, to a certain extent, they strive for shared pursuits (Fitzsimons et al., 2015).

Despite the interdependence of goal striving processes in romantic partners (Rusbult & Van Lange, 2003, 2008), most previous research of goal content has focused only on the individual (Messersmith & Schulenberg, 2010; Ranta et al., 2014; Salmela-Aro et al., 1993, 2007). To date, little is known about the relational dynamics of different personal goal content domains: how partners cooperate and have conflicts around their important projects of various content types. This study aims to understand how different types of personal goals, representing important life domains (cf., Little, 2006) are connected to cooperation and conflict experiences of romantic partners living together. Below, we outline the broader developmental context of goal setting and its effects on goal content. Then we focus on the context of romantic relationships and the role of cooperation and conflict in personal goal accomplishment.

### Developmental context of personal goals in adulthood

Previous research on goal content mainly followed two broad conceptual approaches. The first one is a theory-based approach that categorizes goals according to their assumed motives or higher-order categories, such as implicit motives (Emmons & McAdams, 1991), agency, and communion (Sheldon & Cooper, 2008), and extrinsic versus intrinsic goals (Kasser & Ryan, 1996). Following this theory-based tradition, the Selective Optimization with Compensation (SOC) Model provides a comprehensive conceptualization of personal goals’ developmental context (Baltes, 1987; Baltes & Baltes, 1990; Baltes & Smith, 2004; Freund & Baltes, 2000). Beginning with young adulthood, when people have many biological and social resources available, optimal adaptation involves the maximization of these resources and the orientation towards growth (i.e., higher level of functioning). However, as people grow old, their physical health begins to decline. They become less flexible, and their social environment narrows - consequently, their focus shifts to maintenance in middle adulthood (i.e., stability in functioning). Later on, prevention and compensation of losses prevail in late adulthood (i.e., functioning at a lower level when maintenance is impossible).

The other tradition of goal content research follows a data-driven approach, which aims to categorize goals into specific life domains empirically. Although researchers use different numbers of goal domains in their classifications, their results complement the theoretical assumptions. In support of the SOC model, young adults more frequently reported goals to achieve higher levels of functioning, while middle-aged people strived to ensure the stability of their settled functioning levels (Ebner et al., 2006; Villar & Villamizar, 2012). More specifically, people between the ages of 26 and 34 mention more education and family-related projects, while respondents between 35 and 44 are more concerned about home-related activities than are younger and older age groups (Salmela-Aro et al., 1993). Multiple studies have shown that education-related goals and concerns decrease from the beginning to the end of the second decade of life, whereas engagement in goals related to work and, later, to family and health increases (Ranta et al., 2014; Salmela-Aro et al., 2007).

Gender differences of goal content showed inconsistent results. In a sample of managers, women mentioned more education-related goals, but there was no gender difference with regard to family goals (Salmela-Aro et al., 1993). In a sample of students, men mentioned more daily life- and leisure-related goals than women, while women reported more duty goals than men. Women also showed a steeper increase in child-related goals over a ten year period (Salmela-Aro et al., 2007). Beyond gender, previous commitments, actual life situation and life transitions proved to be more impactful in directing goal-setting, as cohabitating or married students mentioned family-related goals more often than single students (Salmela-Aro & Nurmi, 1997).

To summarize, young adulthood is the time to explore their possibilities and later commit themselves to a chosen career (Super, 1980), romantic partner and friendships (Arnett, 2000; Erikson, 1950). After the main directions to their lives have been established, middle adults strive for stabilization in their chosen profession (Super, 1980), focus on their family and their children’s future while being productive in their social and civic activities (Erikson, 1950; Havighurst, 1980). With higher age, committed relationships become an important developmental task to attain and one of the most influential ties an individual might have (Birditt & Antonucci, 2012). Marriage and other forms of committed relationships can be understood as a permanent social context. This context shapes how partners define and pursue their developmentally relevant personal growth goals at a younger age and then shift their focus to optimal functioning in middle adulthood (Li & Fung, 2011).

### Goal Support and Conflict in Romantic Relationships

As partners get more interdependent during their relationship, their actions and strivings mutually affect each other, which drives both partners to coordinate and align their actions (Rusbult & Van Lange, 2003, 2008). It has been proposed that the goals of relationship partners become so intertwined that their goal-striving process should be better understood when analyzed as one self-regulating system (Fitzsimons et al., 2015). For example, when a husband decides to study coding, he will have limited time to spend with his wife, who, in turn, might be less supportive of his education. The two partners need to negotiate their needs to maintain a well-functioning relationship.

The association between the partners’ level of cooperation and relationship experiences depends on the content of their personal goals. Regarding the similarity of partners’ goals, spouses should see each other as more helpful when their goals align, which, in turn, should be associated with less conflict. Supporting this notion, goal similarity was found to predict goal progress for both relationship goals (Avivi et al., 2009) and academic goals (Fitzsimons & Anderson, 2011). In the long run, couples who coordinate their efforts less efficiently are more prone to divorce, even when controlling for the relationship’s internal quality and several demographic and individual variables (Gere et al., 2016). Thus, partners with different goals might find their everyday interactions more draining and difficult to manage, which can be either a cause or a result of conflicts goals (Fitzsimons & Anderson, 2011). Partners in developing romantic relationships tend to downgrade and eventually drop personal goals when they feel that these goals conflict with their partner’s strivings.

Beyond these general associations, there has been little scholarly focus on the specific contents of personal goals in a relationship, with some notable exceptions. Meegan and Goedereis (2006) found that partners more often classify life tasks concerning relationship, health, leisure, spirituality and finances as interdependent. In contrast, education and work-related life tasks are more frequently considered individual. Couples were more involved in their partner’s interdependent life task pursuit, which, in turn, is connected to daily positive affect. Salmela-Aro et al. (2010) examined the role of spousal support for different kinds of personal goals in a sample of pregnant women. The women received more spousal support for their shared goals (consisting of family, childbirth and motherhood) than for their self and achievement-related goals, which were considered more individualistic. Consequently, goal support predicted relationship satisfaction in the later periods of pregnancy.

### The Present Study

There is growing evidence that joint goal pursuit (or the lack thereof) is an important factor for romantic couples regarding their goal progress (Avivi et al., 2009; Fitzsimons & Anderson, 2011; Sadikaj et al., 2015). Having a committed romantic relationship is not only an essential aspect of goal-related behaviors but achieving and maintaining it can also be considered as an age-graded developmental task per se (Erikson, 1950; Salmela-Aro et al., 2007). Although there is growing interest in incorporating the effect of interpersonal relations into theorizing and research (Fitzsimons et al., 2015; Oishi & Graham, 2010), most previous research on goal content has adopted an individualistic approach (Messersmith & Schulenberg, 2010; Ranta et al., 2014; Salmela-Aro et al., 1993, 2007). In contrast, the few studies that took the role of the romantic partner into account involved small (Meegan & Goedereis, 2006) or specific samples (Salmela-Aro et al., 2010).

The present study aims to bridge the gap of investigating different types of goal domains embedded in the dynamics of romantic relationships. To this end, we used Personal Projects Analysis since it provides a personally meaningful measurement of goals linked to behavior in their context (Little, 2015). Scant research has focused on the contents of personal projects in the literature; most such studies examined a specific target group (managers in the study by Salmela-Aro et al. (1993) and young adults in the work of Ranta et al. (2014)). Despite this scarcity, the number of life domains is quite heterogeneous in the literature, ranging up to 18 (i.e., Salmela-Aro & Nurmi, 1997). To extract a smaller number of meaningful Personal Project Content Domains (PP Content Domains), first, we explored the individual types of personal goal orientations using a pattern-oriented approach (Bergman & Trost, 2006). Although our approach is exploratory, we expect learning, work, relationships, health and instrumental maintenance as emerging central domains, based on previous data (Ranta et al., 2014; Salmela-Aro et al., 1993, 2007).

Second, we aimed to link the resulting PP Content Domains to relationship experiences, namely, perceived cooperation and conflict. We used a relationship-focused analysis to determine which types of goals might be beneficial or detrimental to the relationship. Using this approach, we can unravel not only how the different orientations relate to one’s experience of conflict and cooperation but also to the partner’s experience of conflict and cooperation. The main question is whether specific types of goals connected to life domains might benefit the relationship by facilitating cooperation. Conversely, are there types of goals whose pursuit is more threatening to the relationship because of their association with conflict in the couple? Given that we have broad assumptions about the resulting PP Content Domains at this point, we can only draw general hypotheses. Based on previous literature (Meegan & Goedereis, 2006; Salmela-Aro et al., 2010), we expect more interdependent PP Content Domains (presumably, relationship and health) to associate with more perceived cooperation and less perceived conflict.

## Methods

### Procedure

Ethical approval from Semmelweis University IRB (SE TUKEB) was acquired before the data assessment. Potential participants were contacted through experienced research assistants of the survey firm in their homes. Informed consent was obtained from both partners. The consent form declared that participants were free to withdraw from the study at any time. It also included a short section indicating that the respondents could contact the research team if they needed professional help. The contact information of the PI (the last author), who has extensive counseling experience and who could have referred participants to appropriate resources, was provided on the consent form. No such request was received. The interviewer administered the questionnaire pack to each participant. The assessment procedure was completed by the participants themselves, where spouses were explicitly instructed to work separately. All the provided data were managed confidentially. Inclusion criteria for the couples included a) at least one year of cohabitation, b) at least one partner with a job, and c) no psychiatric episodes in the last 5 years. The partners participated voluntarily in the study and received a book voucher for their contribution (6000 HUF, ~US$20 per couple). Data was collected in 2013–2014 as part of a larger study.

### Sample

Participants were 270 heterosexual couples from Hungary. Twenty-one of them were excluded from the analysis due to invalid or incomplete responses given by at least one of the partners or because they did not meet the criterion of at least one year of cohabitation.

Descriptive statistics of the participants are presented in Table 1. Mean age was 42 years for men (SD = 10.76) and 39.64 years for women (SD = 10.21). The average relationship length was 15.64 years (SD = 9.68 years). Regarding relationship status, 108 couples (43.4%) were cohabiting, and 141 were married (56.6%). Cohabiting couples were significantly younger (m = 38.52 years, SD = 11.14 years for men and m = 36.88 years, SD = 10.62 years for women) and lived together for a shorter time (m = 8.56 years, SD = 5.58 years) than did married couples (m = 45.1 years, SD = 9.51 years for men and m = 42.46 years, SD = 9.09 years for women; m = 19.21 years, SD = 9.34 years for the relationship, respectively). 23.39% of the participants had earned college diplomas (35.34% of men and 22.76% of women), 39.52% held high school diplomas (44.18% of men and 39.18% of women) and 37.1% had completed only primary education (20.48% of men and 38.06% of women).

**Table 1:** Sociodemographic description of the sample

		Cohabiting					Married				
		N	m	SD	Min	Max	N	m	SD	Min	Max
Age	M	109	38.046	11.089	22	67	140	54.071	9.448	30	67
	F	108	35.963	10.542	21	65	141	42.461	9.01	27	66
Number of Children	M	109	0.743	1.013	0	4	140	1.864	1.146	0	7
	F	102	0.706	1.021	0	4	140	1.829	0.981	0	5
Subjective financial status	M	104	5.462	2.033	1	10	137	5.307	1.789	0	9
	F	108	5.157	2.128	0	10	140	5.243	1.815	0	9
Length of relationship (in years)		69	8.551	5.573	3	31	137	19.321	9.378	3	44

### Measures

#### Demographic Variables

Participants completed a demographic information section that included questions on gender, age, education, relationship status, relationship length, number of children and subjective financial status rated on a 11-point scale (from 0 = very bad to 10 = very good).

#### Personal Project Assessment

Partners individually filled in a revised version of Little's (1983) Personal Project Assessment procedure. In the first step, participants were asked to write a list of their personally relevant projects, defined as follows: “the goals and strivings that you are currently working on in your everyday life.” In the second step, respondents selected the four most relevant personal projects from the list. Finally, they were asked to rate each of the four projects along several predefined aspects using a 7-point Likert type scale. These included questions about their individual and relationship focused experiences regarding their projects, including cooperation (How frequently do you cooperate with your partner on this project?) and conflict (How frequently do you have conflicts with your partner over this project?). The other dimensions and results are not reported here. The four project ratings were then aggregated into one score for cooperation and conflict separately, which represents a general experience related to goal cooperation and goal conflict in the relationship.

#### Content analysis of Personal Projects

Each selected and rated goal was classified by two independent evaluators into one of seven categories on the basis of content. The categorization followed the guidelines given by Little and Gee (2007): interpersonal (“spend more time with my family”), academic (“language learning”), work (“better position at work”), intrapersonal (“more order to my life”), recreational/leisure (“holiday”), health (“lose some weight”) and maintenance (“buy a new car”). The responses also contained several skill-oriented learning goals (“get a driver’s license”) that can be acquired in non-academic settings or through self-development. In order to maintain the original categories of the guidelines (Little & Gee, 2007) while incorporating the broader meaning in the present study, these projects were labeled as ‘academic/learning.’

The cross-rater reliability of the ratings measured by the κ was .842, indicating good initial agreement between the content evaluations. The differences were resolved by discussion, and the personal project contents were accounted for according to the final agreements.

For each participant, the total number of personal projects coded in each content category was summarized. Because the resulting distribution of the summarized content frequencies was highly positively skewed (the majority of the data was 0 or 1), the data was recoded into a three-level variable where 0 means that type of content was not mentioned at all, 1 means it was mentioned once and 2 was coded for multiple mentions of the same category. The resulting frequency of mentioned personal project categories is presented in Table 2.

**Table 2:** Frequency of personal project categories

	Number of projects											
	Male partners						Female partners					
	0		1		2–4		0		1		2–4	
	N	%	N	%	N	%	N	%	N	%	N	%
Interpersonal	105	42.17	108	43.37	36	14.46	91	36.55	112	44.98	46	18.47
Academic/learning	192	77.11	45	18.07	12	4.82	151	60.64	78	31.33	20	8.03
Work	108	43.37	124	49.80	17	6.83	137	55.02	104	41.77	8	3.21
Intrapersonal	237	95.18	11	4.42	1	0.40	236	94.78	12	4.82	1	0.40
Recreational / Leisure	154	61.85	87	34.94	8	3.21	159	63.86	87	34.94	10	4.02
Health	174	69.88	69	27.71	6	2.41	150	60.24	84	33.73	15	6.02
Maintenance	44	17.67	75	30.12	130	52.21	65	26.10	91	36.55	93	37.35

N = 249 for male and female partners proportions (%) are given for a specific content domain within male and female partners

### Overview of the Analytical Process

First, to better understand the internal organization of the projects, we subjected the content categories to cluster analysis (for a similar procedure see Marttinen & Salmela-Aro, 2012) using the pattern recognition model of ROPstat software (Vargha, 2016). The appropriate number of clusters was identified through an iterative process (c.f., Takács et al., 2015; Vargha et al., 2016).

To explore how the different PP Content Domains relate to the perceived frequency of cooperation and conflict of personal projects in the context of a romantic relationship, data were analyzed using the Actor-Partner Interdependence Model (Kenny, 1996). The APIM was developed to measure the bidirectional effects in interpersonal relationships (Cook & Kenny, 2005), while controlling for the covariance and statistical dependency that naturally emerges among dyads, and is a generally applied method for analyzing dyadic data (Kenny, 2018). The clusters of life domains were regarded as predictors in the model, while perceived cooperation and conflict were considered as outcomes in two separate models. The actor effect signifies how well the respondents’ project contents predict their own perceived cooperation and conflicts while the partner effect indicates how one’s project content contributes to the cooperation and conflict perceived by the partner. All APIM analyses were calculated using the MIXED procedure in SPSS (SPSS Version 24). Actor and partner effects are both reported as regression coefficients.

## Results

### Preliminary Analyses

First, the reliability of the aggregated ratings of personal projects (cooperation and conflict) was tested. Because of the multilevel structure of the data, intraclass correlation was calculated as a measure of reliability (Lüdtke & Trautwein, 2007). All scales were sufficiently reliable (cooperation ICC (2) = .753 and .655; conflict ICC (2) = .562 and .633 for men and women respectively). The basic psychometric properties and bivariate associations for the study variables were computed (see Table 3). The association on the same scales between the two genders are of medium effect size (r = .436, *p* < .001 for cooperation and r = .464, *p* < .001 for conflict), which indicates dyadic interdependence. APIM analysis is thus required for proper inspection of the relationship outcomes. The correlation between the two different scales was modest for male partners (r = .154, *p* < .05) while there was no significant correlation for female partners (r = .019, *p* > .05).

**Table 3:** Descriptive statistics, psychometric properties and bivariate correlations for the variables

					Pearson correlation coefficients			
		Range	m	SD	1	2	3	4
1	Cooperation Male	1–7	4.85	1.25	.633			
2	Cooperation Female	1.75–7	4.81	1.207	.436**	.562		
3	Conflict Male	1–7	2.58	1.39	.154*	.032	.753	
4	Conflict Female	1–6.5	2.31	1.16	.098	.019	.464**	.655

Reliability estimates (ICC) in the diagonal; * *p* < .05; ** *p* < .01; N = 249

### Cluster Analysis of the Contents of Personal Projects

A series of hierarchical cluster analysis was conducted on the content categories of Personal Projects using the Ward method with squared Euclidean distances. Following the procedure described by Vargha et al. (2015) the adequacy of 2–10 cluster solutions was compared. The main adequacy measures are presented in Table 4 for cluster solutions with 2–10 clusters.

**Table 4:** Adequacy indexes of cluster solutions 2–10

Step	Cluster N	EESS %	Point biserial	XieBeni (mod)	Silhouette coefficient	HC mean
i = 488	10	59.7	0.373	0.507	0.51	0.297
i = 489	9	57.53	0.374	0.392	0.487	0.312
i = 490	8	54.85	0.375	0.133	0.472	0.331
i = 491	7	51.95	0.375	0.197	0.466	0.352
i = 492	6	48.86	0.405	−0.308	0.434	0.374
i = 493	5	45.15	0.51	0.103	0.495	0.4
i = 494	4	40.37	0.494	0.464	0.534	0.434
i = 495	3	34.35	0.439	0.306	0.542	0.477
i = 496	2	22.96	0.325	0.182	0.549	0.558
After relocation	4	42.17	0.51	0.374	0.553	0.421

EESS = Explained Error Sum of Squares; Point biserial = point biserial correlation coefficient; XieBeni (mod) = modified Xie-Beni index; HC = Homogeneity of Cluster index

Determination of the final number of clusters was based on inspection of the adequacy measures and interpretability of the potentially well-fitting cluster solutions. As a result, the four-cluster solution was retained because it had both a satisfactory goodness of fit and offered the best interpretability. Comparing the adequacy measures, the *N* = 4 cluster solution appears to be appropriate in several ways. First, explained variance (EESS = 40.37%) is acceptable because it might be expected to increase after relocation (Vargha et al., 2016). Second, the point biserial coefficient is far above the 0.3 threshold, almost reaching 0.5. Finally, the modified Xie-Beni index indicated a local maximum, with the second largest after the ten-cluster solution. Given that it would be quite challenging to interpret the ten-cluster solution, and that it would contain a small sample size of cluster members, the four-cluster solution was evaluated as a better option in this regard. Moreover, the four-cluster solution was the most homogenous compared with its neighbors. Therefore, the four-cluster solution was retained for further analysis. To make the individual cases match their final cluster better, a relocation process was performed, which resulted in an increased EESS % to 42.17, and the modified Xie-Beni index remaining above 0.3. The individual cases were assigned to their relocated clusters for further analysis. The proportions of the final clusters are well balanced for females, ranging from 12.45% to 33.73%, and a slightly higher deviation for males, ranging from 8.43% to 49.40%.

**Fig. 1:**
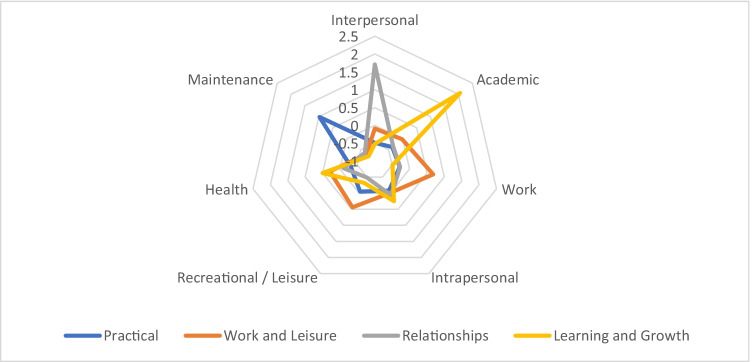
Cluster means of seven personal project content categories in the four cluster solution. Note: Cluster means are z-scores of frequencies

The clusters (i.e., subgroups of individual respondents) that emerged from the four-cluster solution were named after the content category of their most salient personal project variables. The resulting clusters represent PP Content Domains with content category types that were more commonly given by each group member. Inspection of the subgroup means of the initial variables and their graphic representation (Fig. 1) revealed that two clusters, the first and the third, could be characterized by one salient PP Content Domain. The first and largest cluster could be characterized by maintenance goals and, thus, it was named ‘Practical orientation’ cluster. This group included 123 (49.4%) males and 84 (33.73%) females. The third cluster could be characterized by interpersonal goals; correspondingly, it was labeled ‘Relationship orientation’ cluster, representing 36 (14.46%) males and 47 (18.88%) females.

The remaining two clusters had multiple salient PP Content Domains, that is, high subgroup means on the relevant dimensions. In the second cluster, participants mentioned personal projects primarily from two life domains: work, as well as recreation and leisure. It was named ‘Work-Life Balance orientation’ cluster because the main characteristic of this group was the tendency to simultaneously striving for these domains of life. This cluster contained 69 (27.71%) males and 87 (34.94%) females. The fourth and smallest cluster had three salient characteristics, namely academic/learning, health and intrapersonal goals. As mentioned, goals labeled as ‘academic/learning’ represent general practical or skill-oriented learning goals as well. Combined with health and intrapersonal goals, this cluster was labeled ‘Learning and Growth orientation’ cluster, comprising of 21 (8.43%) males and 31 (12.45%) females.

A series of one-way ANOVAs was conducted to examine the differences between the four clusters regarding the sociodemographic background of the participants (see Table 5). The analysis showed significant differences in age [F(3, 248) = 3.57, *p* = 0.02, η^2^ = 0.04 for males and F(3, 248) = 4.41, *p* = 0.01, η^2^ = 0.05 for females], subjective financial status [F(3, 241) = 7.245, *p* < 0.01, η^2^ = 0.08 for males and F(3, 247) = 2.79, *p* = 0.04, η^2^ = 0.03 for females] and length of relationship for females [F(3, 205) = 4.75, *p* < 0.001, η^2^ = 0.07]. There was no significant difference in the length of relationship for the male partners [F(3, 205) = 1.8, *p* = 0.15, η^2^ = 0.03]. Gabriel post hoc analysis showed that males characterized by Learning and Growth orientation are significantly younger than males with Practical orientation (*p* = 0.01). The same difference was seen among females in that both the Practical orientation group (*p* = 0.02) and the Work-Life Balance orientation group was significantly older than the Learning and Growth orientation group, and had a significantly longer relationship (*p* = 0.032 and *p* = 0.013). Learning and Growth orientation group rated their financial status higher compared to Practical orientation for both genders (*p* < 0.001 for males and *p* = 0.023 for females respectively) and compared to Work-Life Balance orientation for males (*p* = 0.012). Males with Relationship orientation also considered their financial status higher than males with Practical orientation (*p* = 0.012). There was no significant association between cluster membership and relationship status (married or cohabitating) [Χ^2^ = 0.638 (3), *p* = 0.888, φ = 0.051 and Χ^2^ = 0.487 (3), *p* = 0.922, φ = 0.044 for male and female partners, respectively] or between cluster membership and raising underage children for females [Χ^2^ = 1.522 (3), *p* = 0.677, φ = 0.079]. A significant association was found between cluster membership and education [Χ^2^ = 27.396 (3), *p* < 0.001, φ = 0.332 and Χ^2^ = 13.338 (3), *p* = 0.038, φ = 0.231 for male and female partners, respectively], and between cluster membership and raising underage children for males [Χ^2^ = 1.17.589 (3), *p* = 0.001, φ = 0.266].

**Table 5:** Descriptive statistics and comparison of PP Contant Domain clusters

Clusters of the content of personal projects												
		Practical		Work-Life Balance		Relationships		Learning and Growth				
		M	SD	M	SD	M	SD	M	SD	F	*p*	η2
Males	Cooperation	4.97	1.24	4.68	1.32	5.31	0.97	4.18	1.10	4.62	< .001	0.05
	Conflict	2.76	1.55	2.45	1.27	2.48	1.36	2.12	1.01	1.63	0.18	0.02
	Age	43.89	10.52	40.78	10.98	41.06	10.69	36.52	9.53	3.57	0.02	0.04
	Length of relationship	16.90	9.68	15.76	10.66	13.16	8.83	12.47	6.98	1.81	0.15	0.03
	Subjective financial status	4.96	2.01	5.36	1.82	6.00	1.47	6.75	1.07	7.25	< .001	0.08
Females	Cooperation	4.99	1.13	4.76	1.21	5.02	1.11	4.13	1.31	4.67	< .001	0.05
	Conflict	2.48	1.26	2.19	1.13	2.34	1.21	1.98	0.97	1.71	0.17	0.02
	Age	41.55	9.97	40.33	10.80	38.55	9.74	34.19	7.91	4.41	0.01	0.05
	Length of relationship	16.92	9.49	17.54	10.83	12.76	7.83	10.96	6.73	4.75	< .001	0.07
	Subjective financial status	4.94	2.06	5.24	1.94	5.04	1.96	5.21	1.95	2.79	0.04	0.03

Finally, the differences among the four clusters regarding the outcome variables were tested. Significant differences were observed for cooperation for both spouses [F(3, 248) = 4.62, *p* < 0.001, η^2^ = 0.05 and F(3, 248) = 4.672, *p* < 0.001, η^2^ = 0.05 for male and female partners respectively]. Applying the Gabriel test to subgroup differences post hoc indicated less cooperation perceived by the Learning and Growth orientation group than the Practical (*p* = 0.02) and Relationship (*p* = 0.004) orientation group for males. Similarly, the Learning and Growth orientation group perceived the least cooperation of any other group among females (*p* = 0.002 for Practical, *p* = 0.048 for Work-Life Balance and p = 0.007 for Relationship orientation, respectively). There were no significant differences among the clusters for both spouses regarding perceived conflict [F(3, 248) = 1.63, *p* < 0.18, η^2^ = 0.02 and F(3, 248) = 1.71, *p* = 0.17, η^2^ = 0.02 for male and female partners, respectively].

For further analysis, cluster membership was dummy coded using Practical orientation, i.e. the largest group, as a reference category. Practical orientation is also a good fit for reference category from a conceptual standpoint, because this label covered partially mundane tasks that can be considered more ad hoc and less of a conscious aspiration (compared with goal orientations regarding Work-Life Balance, Relationships, Learning and Growth). Below, the note to the reference category will be omitted to increase clarity. However, all the results concerning the PP Content Domains should be interpreted in contrast to the occurrence of Practical orientation.

#### PP Content Domains and perceived cooperation and conflict in romantic dyads

**Fig. 2:**
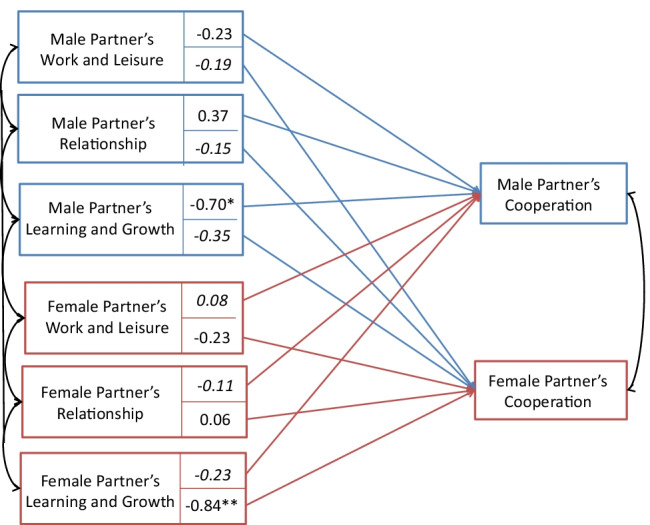
The APIM model showing the intercepts of actor and partner effects for perceived cooperation in PP Content Domains. Note: * *p* < .05; ** *p* < .001

Two main APIM models were tested for the two relationship outcomes separately. First, the contribution of PP Content Domains contents to cooperation was tested (see Table 6 and Fig. 2). The three dummy-coded PP Content Domain variables (Work-Life Balance, Relationships, Learning and Growth compared with Practical orientation) were regressed on perceived cooperation related to personal projects. Learning and Growth orientation was associated with lower perceived cooperation in their own personal projects by both spouses (*β* = −0.70, *p* = 0.02 for males and *β* = −0.84, p < 0.001 for females). The other PP Content Domains have no significant effect on cooperation in personal projects.

**Table 6:** Personal Project Content Domains predicting male and female partners’ perceived cooperation

		Male partners’ cooperation experiences			Female partners’ cooperation experiences		
		*β*	*t*	*p*	*β*	*t*	*p*
Male partner	Work-Life Balance orientation	−0.23	−1.21	0.23	0.08	0.43	0.67
	Relationship orientation	0.37	1.49	0.14	−0.11	−0.46	0.65
	Learning and Growth orientation	−0.7	−2.36	0.02	−0.23	−0.81	0.42
Female partner	Work-Life Balance orientation	−0.19	−1	0.32	−0.23	−1.26	0.21
	Relationship orientation	−0.15	−0.63	0.53	0.06	0.27	0.79
	Learning and Growth orientation	−0.35	−1.33	0.18	−0.84	−3.28	< 0.001

Note. n = 249 heterosexual couples; Results of the APIM regression model Actor and partner effects are reported as regression coefficients

**Fig. 3:**
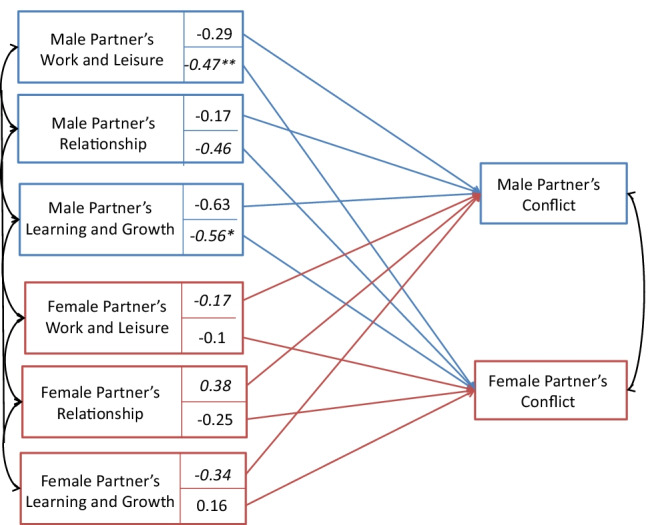
The APIM model showing the intercepts of actor and partner effects for perceived in PP Content Domains. Note: * *p* < .05; ** *p* < .001

In the second model, the three dummy-coded PP Content Domain variables (Work-Life Balance, Relationships, Learning and Growth compared with Practical orientation) were regressed on perceived conflict related to personal projects (see Table 7 and Fig. 3). Two partner effects were identified. Females with a male partner who had Work-Life Balance (*β* = −0.47, *p* = 0.01) or Learning and Growth orientation (*β* = −0.56, *p* = 0.047) perceived less conflict regarding their own personal projects.

**Table 7:** Personal Project Content Domains predicting male and female partners’ perceived conflict

		Male partners’ conflict experiences			Female partners’ conflict experiences		
		*β*	*t*	*p*	*β*	*t*	*p*
Male partner	Work-Life Balance orientation	−0.29	−1.31	0.19	−0.47	−2.64	0.01
	Relationship orientation	−0.17	−0.58	0.56	−0.46	−1.91	0.06
	Learning and Growth orientation	−0.63	−1.83	0.07	−0.56	−2.00	0.05
Female partner	Work-Life Balance orientation	−0.10	−0.43	0.67	−0.17	−0.96	0.34
	Relationship orientation	−0.25	−0.89	0.37	0.04	0.17	0.87
	Learning and Growth orientation	0.02	0.05	0.96	−0.34	−1.37	0.17

Note. n = 249 heterosexual couples

Results of the APIM regression model. Actor and partner effects are reported as regression coefficients.

For the final analysis, demographic control variables (age and length of relationship as centered variables, relationship status (married or cohabitating), raising underage children, levels of education (as dummy coded variables) and subjective financial status were introduced in the models (see Tables 8 and 9). There was no significant association between the demographic variables and perceived cooperation in personal projects. Still, compared to the previous results Learning and Growth orientation was significantly associated with less perceived cooperation in personal projects (*β* = −0.7094, *p* < 0.01) only in the case of female partners. The association between Learning and Growth orientation and perceived cooperation was no longer significant for male partners (*β* = −0.51, *p* = 0.13).

**Table 8:** Results of the APIM regression model involving male and female partners’ perceived cooperation in PP Content Domains with demographic control variables. Actor and partner effects are reported as regression coefficients

		*β*	*t*	*p*
Relationship status (married or cohabitating)		0.05	0.23	0.82
Raising underage child/children		0.12	0.60	0.55
Primary education only vs. high school diploma		0.06	0.40	0.69
College diploma vs. high school diploma		−0.16	−1.21	0.23
Age		0.12	1.22	0.22
Length of relationship		−0.10	−0.91	0.37
Subjective financial status		0.02	0.61	0.54
Male actor	Work-Life Balance orientation	0.01	0.05	0.96
	Relationship orientation	0.39	1.47	0.14
	Learning and Growth orientation	−0.51	−1.54	0.13
Female actor	Work-Life Balance orientation	−0.23	−1.15	0.25
	Relationship orientation	−0.04	−0.18	0.86
	Learning and Growth orientation	−0.94	−3.21	<0.01
Male partner	Work-Life Balance orientation	0.25	1.25	0.21
	Relationship orientation	−0.17	−0.64	0.52
	Learning and Growth orientation	0.13	0.39	0.70
Female partner	Work-Life Balance orientation	−0.18	−0.90	0.37
	Relationship orientation	−0.26	−1.02	0.31
	Learning and Growth orientation	−0.28	−0.96	0.34

**Table 9:** Results of the APIM regression model involving male and female partners’ perceived conflict in PP Content Domains with demographic control variables. Actor and partner effects are reported as regression coefficients

		*β*	*t*	*p*
Relationship status (married or cohabitating)		0.15	0.69	0.49
Raising underage child / children		0.40	1.88	0.06
Primary education only vs. high school diploma		0.18	1.08	0.28
College diploma vs. high school diploma		0.17	1.15	0.25
Age		−0.11	−1.06	0.29
Length of relationship		0.01	0.09	0.92
Subjective financial status		−0.11	−2.94	<0.01
Male actor	Work-Life Balance orientation	−0.13	−0.54	0.59
	Relationships orientation	0.08	0.23	0.81
	Learning and Growth orientation	−0.37	−0.89	0.37
Female actor	Work-Life Balance orientation	−0.17	−0.84	0.40
	Relationships orientation	0.03	0.11	0.91
	Learning and Growth orientation	−0.21	−0.71	0.48
Male partner	Work-Life Balance orientation	−0.42	−2.10	0.04
	Relationships orientation	−0.39	−1.44	0.15
	Learning and Growth orientation	−0.48	−1.38	0.17
Female partner	Work-Life Balance orientation	−0.04	−0.16	0.88
	Relationships orientation	−0.31	−0.98	0.33
	Learning and Growth orientation	0.19	0.53	0.60

Regarding conflict, subjective financial status showed a significant association with conflict, whereas lower subjective financial status increased the probability of perceived conflict in personal projects (*β* = −0.94, *p* < 0.01). There was no significant association between all the other demographic variables and perceived conflict in personal projects. Only the male partner effect for Work-Life Balance (*β* = −0.42, *p* = 0.04) on perceived conflict remained significant, while male Learning and Growth orientation (*β* = −0.48, *p* = 0.17) no longer had a significant effect on female partners’ perceived conflict in personal projects.

## Discussion

In the present study, we examined how different types of goal orientations are embedded in the romantic relationships of adult couples. Using a person-oriented approach, we first extracted four meaningful goal orientation clusters from content domains of personal projects: Practical, Work-Life Balance, Relationships, and Learning and Growth orientations. We then connected these PP Content Domains to the couple’s perceived cooperation and conflict regarding their goal pursuit. Specific relationship outcomes accompanied Learning and Growth orientation and Work-Life Balance orientation. Both partners perceived lower cooperation if they had Learning and Growth orientation goals. In contrast, male partners’ Work-Life Balance or Learning and Growth orientation resulted in less perceived conflict by their female partners regarding their own goals. Below, the potential implications and consequences will be discussed.

### Content Domains of Personal Projects in Romantic Partners

Through cluster analysis, four clusters of personal level goal orientation were identified. The groups were named after their distinctive salient characteristics: Practical, Work-Life Balance, Relationships, and Learning and Growth orientations. As expected, learning, maintenance, work, and relationships emerged as central domains, found as typical projects of young to middle adults in previous studies as well (Ranta et al., 2014; Salmela-Aro et al., 1993, 2007). Moreover, the salient PP Content Domains of our clusters mirror the age-related change of growth to maintenance orientation of personal goals, as proposed by the SOC model (Baltes, 1987; Baltes & Baltes, 1990; Baltes & Smith, 2004; Freund & Baltes, 2000).

Thus, the projects of the Learning and Growth oriented adults, being the youngest subgroup in the sample with the highest subjective financial status, were related to their personal development with a focus on academic or learning, health and intrapersonal goals. This cluster also encompasses the developmental tasks of young adults to explore their options and possibilities (Arnett, 2000). Goals focusing on development, delineation, and higher levels of functioning are common among younger adults in general (Ebner et al., 2006; Villar & Villamizar, 2012). Educational goals were the top priority in previous research done with young adults (Ranta et al., 2014) and college students (Blais et al., 1990). They were mentioned more frequently than in older age groups (Salmela-Aro et al., 1993).

The older and largest cluster, which rated their financial status the lowest, was Practical orientation, which was mainly connected to the pursuit of maintenance projects and captured a developmentally significant shift of focus. This group represents an already settled generative stage of life (Erikson, 1950). In concordance with this interpretation, middle-aged people reportedly aimed to ensure the stability of their life (Ebner et al., 2006; Villar & Villamizar, 2012).

The Work-Life Balance orientation group was, in respect of women partners, older as well, while men rated their financial status relatively low. The cluster’s main characteristic was balancing between two equally salient domains of life: work and recreation or leisure. Balancing multiple challenges might be an aspect of a more settled generative life (Erikson, 1950). People aim for stability in their workplace (Super, 1980), while leisure time with their family might be considered of equal importance.

Finally, the Relationship orientation cluster was primarily related to interpersonal projects. This cluster was the most diverse group concerning their sociodemographic characteristics, such as age, length of the relationship, education and raising underage children. Even though the family was not distinguished from other types of social relationships in our study, this finding contrasts with the previous results with family goals prominent only in middle adulthood (Ranta et al., 2014; Salmela-Aro et al., 1993, 2007). However, in romantic couples living together, Relationship orientation may be salient, regardless of their age and relationship status. For these couples, their relationship might be considered a permanent framework through which the other type of goals can be realized (Li & Fung, 2011).

### Content Domains in Romantic Relationships

As for the second objective for this study, in analyzing the data on the dyadic level, the PP Content Domains were connected to the perceived cooperation and conflict regarding personal projects. Despite our expectations, no PP Content Domains were associated with a higher level of perceived cooperation; in contrast, the perception of less cooperation proved to be more salient for specific life domains.

Couples with Learning and Growth orientation or Work-Life Balance orientation had unique relationship experiences. The actors’ Learning and Growth orientation was associated with less perceived cooperation in the goals by both male and female partners. Female partners whose spouses had Work-Life Balance or Learning and Growth orientation perceived less conflict regarding their own goals. In other words, both partners seek less cooperation when they have Learning and Growth orientation goals, and women with Learning and Growth oriented or Work-Life Balance oriented partners perceive less conflict regarding their projects.

The results are consistent with previous research on married couples’ life tasks (Meegan & Goedereis, 2006). Work and education life tasks were classified as more individual as compared to relationship, health, leisure, spirituality and financial life tasks, that were rated more interpersonal. In turn, interdependent life tasks lead to more spousal involvement on a day-to-day basis. However, depending on how well the two partners coordinate their goals, greater interdependence might be a double-edged sword. Greater interdependence might raise opportunities to facilitate and obstruct each other’s goal pursuit (Fitzsimons et al., 2015). Future research could specifically assess couples’ interdependence and elaborate further whether this variable plays a moderating role in the associations between goal orientations and conflict or cooperation.

Our results are coherent with Villar and Villamizar's (2012) study, which found a negative association between relationship satisfaction and goals oriented towards gains among all older age groups (over age 30). They argued that the couple relationship itself might be so fundamental that aspiring developmental-graded gains might no longer contribute to marital satisfaction once it is achieved. Emphasizing maintenance and keeping the bond safe becomes more substantial for relationship satisfaction. This might also suggest a time window when goals related to individual growth are supposed to be realized. Prolonging or resetting these types of goals later might remove valuable resources from the couple’s joint pool, which might threaten their shared interpersonal goals, including their relationship.

Overall, Learning and Growth oriented goals can be considered more distant from the dynamics of romantic relationships in that they involve less joint experiences, cooperation and conflict. When analyzed on a personal level, Learning and Growth orientation was more common among younger adults. Consequently, one might argue that less perceived goal coordination and conflict result from a younger age and a less engaged relationship in general. However, when analyzed on a dyadic level, age did not affect the whole model. When we incorporate all the demographic variables into the models, only subjective financial status increased the perceived conflict in the goals at the edge of significance. There were no correlations between any other demographic variable and perceived cooperation, including age, length of relationship, relationship status, levels of education and having children. Results confirm that economic strain can induce negative interaction between the couple (Falconier & Jackson, 2020), and imply that financial stress might be an influential context for the couple’s functioning beyond the content of their goals.

### Limitations

While evaluating the results of this study, some limitations should be considered. First, we conducted cross-sectional research; therefore, causal explanations cannot be concluded. Only a longitudinal study could more precisely discern the (possibly circular) connection between the partners’ different goal contents and their relationship dynamics. Second, we relied solely on self-reported responses and avoided using preexisting, standardized scales, which might have limited the generalizability of our results. Third, there is a possible bias in the investigation since the sample was not representative, and social desirability was not controlled for in the analyses. Fourth, given that our sample consisted only of Hungarian couples, cross-cultural reliability of the PP Content Domains might be important for future research. Fifth, beyond the demographic control variables we used in our model, there might be additional relevant background variables detailing the couple’s relationship (e.g., the couple’s development, the partners’ breakup history and attachment patterns) and their broader socioeconomic context (e. g., the age of the children and their health status, the couple’s employment-status). These aspects of the relationship may be addressed in future research. Sixth, data was collected before the COVID-19 pandemic, which might limit the current generalizability of our results. Lastly, we used the aggregated evaluations from all four personal projects, which weakens the link between the content category and the perceived cooperation and conflict. Later studies might evaluate and compare predefined goal domains.

## Conclusions

Our results shed light on life domains reflected in the content of personal projects and provide insight into how well these life domains fit into the dynamics of romantic relationships. It adds to our knowledge on cooperation and conflict romantic couples perceive in their different types of personal goals. Our main result is that Learning and Growth-oriented goals remain the furthest from the dynamics of the relationship because they invoke less cooperation for both partners and less conflict in the individual’s own goals in the case of women with Learning and Growth-oriented partners.

Moreover, our results have specific implications for praxis. Couples should be attentive when one of the partners sets a Learning and Growth-oriented goal, especially when the partners are approaching or have reached middle adulthood and have obligations at work and towards their family. Engaging in interdependent projects, partners may improve their goal coordination and find ways to negotiate conflicts constructively. These efforts may lead to share their resources and protect their bond.

## Data Availability

The datasets analysed during the current study are available from the corresponding author on reasonable request.
